# Investigating the association between urban rural classification with fragility fractures and bone density in the UK: evidence from a large observational clinical cohort

**DOI:** 10.1093/rap/rkag004

**Published:** 2026-01-08

**Authors:** Hamzah Amin, Muhammed Aqib Khan, Marwan Bukhari

**Affiliations:** Faculty of Health and Medicine, Lancaster University Medical School, Lancaster University, Lancaster, UK; Department of Rheumatology, University Hospitals of Morecambe Bay NHS Foundation Trust, Lancaster, UK; Faculty of Health and Medicine, Lancaster University Medical School, Lancaster University, Lancaster, UK; Department of Rheumatology, University Hospitals of Morecambe Bay NHS Foundation Trust, Lancaster, UK

**Keywords:** urban rural, population health, geography, bone density, fragility fracture

## Abstract

**Objectives:**

Geographic variation in fracture risk exists across the UK, yet rural/urban differences in fracture risk and bone density have yet to be explored. We aimed to investigate associations between rural urban classification (RUC) and bone health outcomes in a UK clinical population.

**Methods:**

We analysed patients who underwent DXA scanning between June 2004 and May 2025 in northwest England. Geographic status was assessed using the 2011 UK Census RUC. Multiple imputation with parameter pooling was performed with logistic and linear regression models adjusted for Fracture Risk Assessment Tool (FRAX) risk factors to investigate the associations between urban and rural residence and major osteoporotic fractures, hip fractures, bone mineral density and body composition.

**Results:**

Of 40 951 eligible patients, 32 324 (79%) were women with a mean age of 68.2 years; 11 811 major osteoporotic fractures were reported including 2208 hip fractures. No significant association existed between rural/urban status and odds of having a previous fragility fracture [major osteoporotic fractures: odds ratio (OR) 0.97 (95% CI 0.92, 1.02); hip fractures: OR 0.98 (95% CI 0.90, 1.07)]. Rural residence was associated with lower osteoporosis odds [femoral neck OR 0.86 (95% CI 0.80, 0.92); lumbar spine OR 0.85 (95% CI 0.80, 0.91)]. Rural patients had significantly lower regional body fat [femoral fat percentage: β = −0.45 (95% CI −0.55, −0.34), *P* < 0.001; abdominal fat percentage: β = −0.43 (95% CI −0.58, −0.29), *P* < 0.001].

**Conclusions:**

Rural residence was associated with improved bone density and body composition but was not associated with fragility fractures in our referred UK clinical cohort.

Key messages

- There was no difference in fragility fracture odds between urban and rural patients.
- Patients in a rural setting had reduced odds of being diagnosed with osteoporosis.
- Patients in a rural setting had less abdominal and femoral fat.

## Introduction

Osteoporosis is a skeletal condition characterized by a reduction in bone mineral density (BMD) ≥2.5 s.d. below the mean of a young, healthy adult population, as measured by DXA [1]. Although asymptomatic, osteoporosis significantly increases the risk of fragility fractures, which are fractures occurring from low-impact trauma, such as a fall from standing height or less [2, 3]. Fragility fractures are associated with increased morbidity and mortality and present a major public health burden [4]. In the UK alone, the estimated annual cost to the National Health Service (NHS) for managing fragility fractures is £4.6 billion, a figure expected to increase with the aging population [5].

Geographical distribution has been recognized as a factor influencing bone health [6]. In low- and middle-income countries (LMICs), urban living has been associated with higher bone mineral density (BMD), likely due to improved nutrition and better access to healthcare services [6]. However, this pattern appears to reverse in high-income countries. For instance, data from the South Dakota Health Study suggest that urban men have higher BMD, although physical activity was not identified as a contributing factor [7]. Other studies have suggested that rural residences are associated with decreased odds of fragility fracture [8], although limited evidence explores urban/rural differences in relation to fracture risk.

In the UK, substantial regional variation in fracture risk has been reported. For example, the fracture incidence in Scotland has been found to be 50% higher than in regions of the south [9]. Furthermore, highly urbanized areas such as London have been reported to have the lowest rates of fragility fractures nationally [9]. Despite these findings, rural populations, who comprise ≈17% of the UK population, remain underrepresented in osteoporosis and fracture risk research [10]. There is a notable lack of studies in the UK exploring the association between rural urban classification (RUC) and bone health outcomes such as BMD, fragility fractures and regional body composition.

The primary aim of this study was to investigate differences in fragility fracture incidence, DXA-derived BMD and DXA-derived regional body composition between individuals residing in urban *vs* rural settings within a large UK clinical cohort.

## Methods

### Study population and data collection

Between June 2004 and May 2025, a total of 46 487 patients were referred for DXA scanning at our centre located in northwest England. The scanner serves a regional population across South Cumbria and Lancashire, which includes a higher proportion of rural residents compared with the national average, estimated at ≈20% [11].

All patients underwent a standardised clinical consultation with a trained clinician as part of their clinical assessment for osteoporosis. This assessment included measurement of height and weight and a structured medical history focused on recognised fracture risk factors. These included current or previous use of glucocorticoids (defined as a prednisolone equivalent dose of at least 5 mg daily for ≥3 months), a history of previous fragility fracture, family history of hip fracture, current smoking status, alcohol consumption of at least 3 U/day and a diagnosis of RA. A detailed fracture history was also obtained to assess both the presence and location of any fragility fractures before the scan date and defined as fractures resulting from low-energy trauma. All reported data were cross verified against each patient’s electronic medical records before entry into the study database.

Most patients underwent DXA scanning of both femoral necks and the lumbar spine, which represents routine clinical practice at our centre. Scans conducted between 2004 and 2019 used the GE Lunar Prodigy machine, while from 2019 onward the GE iDXA system was employed (GE Healthcare, Chicago, IL, USA). At the time of system transition, the devices were cross calibrated to ensure continuity of data quality. Ongoing quality control has been maintained through weekly phantom scans.

Patient demographic data, including residential postcode, were also collected. Postcodes were used to assign each patient to a Lower Super Output Area (LSOA). LSOA codes were then matched with the 2011 UK census RUC [12]. We were unable to locate 2021 census RUC. Furthermore, it was deemed that 2011 RUC data would better align with the temporal range of our dataset, given our data goes back to 2004.

Full ethical approval for pseudonymized data extraction in the absence of informed consent was obtained from the regional NHS Research Ethics Committee Northwest Preston (project 14/NW/1136).

### Statistical analysis

The RUC from the 2011 census categorises areas according to the degree of urbanisation and rurality. For the purposes of this analysis, the variable was dichotomised into urban and rural categories to improve statistical power and interpretability. Areas classified as any type of rural were grouped together, with any urban settings grouped together and serving as the reference group.

Only patients >50 years of age were included in our analysis. We first compared baseline demographic and clinical characteristics, including Fracture Risk Assessment Tool (FRAX)-based risk factors, between the patients in an urban *vs* rural residence. Continuous variables were compared using the Student’s *t*-test, given normality is assumed in large datasets, while categorical variables were assessed using the chi-squared test. In addition, we also evaluated whether baseline characteristics differed between patients with known urban/rural status and those with missing data using the same statistical tests.

Given the relatively high level of missingness in the RUC variable, as well as missingness in several other key predictor variables, we performed multiple imputation using the miceFast package in R (R Foundation for Statistical Computing, Vienna, Austria), after confirming that the data were missing at random. Five imputed datasets were generated and used for our different analyses (described below). Owing to the high dimensionality of our dataset (≈45 000 participants and 300 variables), miceFast was selected for its computational efficiency. Imputation methods were tailored to the variable type: Bayesian linear regression was applied to continuous variables, predictive mean matching to ordinal variables and linear discriminant analysis to binary and categorical variables.

We then fitted models across the different outcomes, including reporting a previous major osteoporotic fracture (defined as fractures of the spine, proximal femur, humerus and forearm, including the radius and ulna) and hip fracture, which was modelled independently because FRAX provides a separate estimate for hip fracture risk. We also modelled the odds of being diagnosed with osteoporosis at the femoral neck and lumbar spine, defined as a T-score of ≤−2.5, which we treated as a binary outcome. In addition, we also modelled regional body composition, focusing on the left femoral region and the abdominal region.

Logistic regression models were used for binary outcomes, which included previous major osteoporotic fracture, hip fracture, femoral neck osteoporosis and lumbar spine osteoporosis. Linear regression models were used for the regional body composition measures, which looked at the abdominal fat percentage (L1–L4 region) and the left femur. All models were adjusted for age at the time of the scan, sex, current smoking status, excessive alcohol use, current glucocorticoid therapy, RA, family history of fracture, BMI and femoral neck T-score where appropriate (T-score adjustment was not applied in the osteoporosis models). Both the body composition models and the osteoporosis models also included major osteoporotic fracture as an additional potential confounder. These models were fitted across all five imputed datasets and parameter estimates were pooled using Rubin’s rules.

A sensitivity analysis was also conducted, where we repeated all modelling (as described above) using complete cases only.

## Results

### Baseline demographics and risk factors summary statistics

A total of 40 951 patients met the eligibility criteria for the study, with 11 811 previous major osteoporotic fractures reported, including 2208 hip fractures. The sample consisted of 32 324 (79%) female patients with a mean age of 68.23 years (s.d. 9.92). Geographic classification data were available for 29 793 participants, with similar representation from urban (*n* = 14 877) and rural (*n* = 14 916) areas. Across the full cohort, the mean femoral neck BMD T-score was −1.30 (s.d. 1.15) and the mean BMI was 27.15 kg/m^2^ (s.d. 5.42).

Significant differences in bone health were observed between urban and rural populations. Rural participants (*n* = 14 916) demonstrated superior bone density with higher femoral neck (−1.28 *vs* −1.32) and lumbar spine (−0.60 *vs* −0.75) T-scores and correspondingly lower osteoporosis prevalence at both sites (13% *vs* 15% femoral neck, 16% *vs* 18% lumbar spine) (all *P* < 0.001). In contrast, urban participants (*n* = 14 877) had significantly higher BMI (27.35 *vs* 26.96 kg/m^2^), greater body fat distribution (31.56% *vs* 30.57% abdominal fat) and elevated smoking rates (13% *vs* 8.1%) (all *P* < 0.001). Despite these bone density differences, no significant differences were observed between groups in the prevalence of major osteoporotic fractures (31% *vs* 30%; *P* = 0.4) or hip fractures (5.4% *vs* 5.2%; *P* = 0.4). Rural participants were marginally older and more frequently reported corticosteroid therapy use and family history of fracture. Full results are presented in Table 1.

**Table 1:** Comparison of baseline demographics and fracture risk factors between urban and rural patients.

Characteristics	Overall (*n* = 29 793)	Urban [*n* = 14 877 (49.9%)]	Rural [*n* = 14 916 (50.1%)]	*P*-value^a^
Age at scan, years, mean (s.d.)	67.9 (9.9)	67.57 (9.91)	68.30 (9.78)	<0.001
Gender				0.3
Female	24 211 (81.3)	12 122 (81.5)	12 089 (81.0)	
Male	5582 (18.7)	2755 (18.5)	2827 (19.0)	
Major osteoporotic fracture	9053 (30.4)	4552 (30.6)	4501 (30.2)	0.4
Hip fracture	1590 (5.3)	810 (5.4)	780 (5.2)	0.4
BMI (kg/m^2^), mean (s.d.)	27.15 (5.4)	27.35 (5.61)	26.96 (5.22)	<0.001
Current steroid therapy	3465 (11.6)	1652 (11.1)	1813 (12.2)	0.005
RA	2095 (7.0)	1103 (7.4)	992 (6.7)	0.011
Excess alcohol consumption	1758 (5.9)	845 (5.7)	913 (6.1)	0.11
Current smoking	3133 (10.5)	1918 (12.9)	1215 (8.1)	<0.001
Family history of fracture	5689 (19.1)	2772 (18.6)	2917 (19.6)	0.044
Femoral neck BMD T-score, mean (s.d.)	−1.30 (1.15)	−1.32 (1.17)	−1.28 (1.13)	0.001
Missing	1658 (5.6)	777 (5.2)	881 (5.9)	
Lumbar spine BMD T-score, mean (s.d.)	−0.68 (1.97)	−0.75 (1.95)	−0.60 (1.99)	<0.001
Missing	22 (0.1)	16 (0.1)	6 (<0.1)	
Femoral neck osteoporosis (T-score ≤−2.5)	3920 (13.2)	2083 (14.0)	1837 (12.3)	<0.001
Missing	1658 (5.6)	777 (5.2)	881 (5.9)	
Lumbar spine osteoporosis (T-score ≤−2.5)	5038 (17.1)	2669 (17.9)	2369 (15.9)	<0.001
Missing	22 (0.1)	16 (0.1)	6 (<0.1)	
Abdominal fat percentage (%)	31.06 (10.72)	31.56 (10.72)	30.57 (10.70)	<0.001
Missing	2222 (7.5)	1145 (7.7)	1,077 (7.2)	
Left femur fat percentage (%)	30.25 (6.99)	30.74 (7.00)	29.77 (6.95)	<0.001
Missing	3241 (10.9)	1627 (10.9)	1614 (10.8)	

Values are presented as n (%) unless stated otherwise.

a Wilcoxon rank sum test; Pearson’s chi-squared test.

Approximately 11 000 patients had missing geographic data, primarily attributable to incomplete postcode information in the original dataset, with a smaller proportion resulting from linkage errors. Participants with missing urban/rural data (27.2% of the cohort) were slightly older (69.0 *vs* 67.9 years; *P* < 0.001), more likely to be male (27.3% *vs* 18.7%; *P* < 0.001), had a higher BMI (27.8 *vs* 27.1 kg/m^2^; *P* < 0.001) and had a higher regional body fat percentage [abdominal: 32.6% *vs* 31.1% (*P* < 0.001); left femur: 31.0% *vs* 30.3% (*P* < 0.001)] and also differed in clinical characteristics such as RA (5.0% missing *vs* 7.0% available; *P* < 0.001), smoking (2.2% missing *vs* 10.5% available; *P* < 0.001) and family history of fracture (25.2% missing *vs* 19.1% available; *P* < 0.001). Major osteoporotic fracture prevalence was lower in participants with missing geographic data (24.7% *vs* 30.4%; *P* < 0.001). However, imputation was used for our statistical modelling to account for systematic differences in patients with missing data. Detailed comparisons between urban and rural patients are provided in Supplementary Table S1, available at *Rheumatology Advances in Practice* online.

### Fragility fracture models

Multiple imputation with parameter pooling was used for all subsequent models. There was no significant association between RUC and reporting a previous major osteoporotic fracture, with an adjusted OR of 0.97 (95% CI 0.92, 1.02; *P* = 0.212). Similarly, urban/rural status was not significantly associated with previous hip fractures, with an adjusted OR of 0.98 (95% CI 0.90, 1.07; *P* = 0.524). These findings are shown in Table 2.

**Table 2:** Multiple imputation with parameter pooling for fragility fracture models using logistic regression.

Models	Adjusted OR (95% CI)	*P*-value
Major osteoporotic fracture	0.97 (0.92, 1.02)	0.212
Hip fractures	0.98 (0.90, 1.07)	0.524

Models adjusted for age at the time of the scan, sex, current smoking status, excessive alcohol use, current glucocorticoid therapy, RA, family history of fracture, body mass and femoral neck T-score.

### Bone density models

Participants residing in rural areas had significantly lower odds of being diagnosed with osteoporosis at both the femoral neck and lumbar spine compared with urban residents. Specifically, rural residence was associated with an adjusted OR of 0.86 (95% CI 0.80, 0.92; *P* < 0.001) for femoral neck osteoporosis and 0.85 (95% CI 0.80, 0.91; *P* < 0.001) for lumbar spine osteoporosis. These results are shown in Table 3.

**Table 3:** Multiple imputation with parameter pooling for osteoporosis models using logistic regression

Models	Adjusted OR (95% CI)	*P*-value
Femoral neck osteoporosis	0.86 (0.80, 0.92)	<0.001
Lumbar spine osteoporosis	0.85 (0.80, 0.91)	<0.001

Models adjusted for age at the time of the scan, sex, current smoking status, excessive alcohol use, current glucocorticoid therapy, RA, family history of fracture, BMI and major osteoporotic fracture.

### Body composition models

Rural participants had significantly lower femoral fat percentage [β = −0.45 (95% CI −0.55, −0.34)] and abdominal fat percentage [β = −0.43 (95% CI −0.58, −0.29)] compared with urban participants. These results demonstrate that rural residency is associated with a more favourable body composition profile. These results are shown in Table 4.

**Table 4:** Multiple imputation with parameter pooling for body composition models using linear regression.

Models	Adjusted OR (95% CI)	*P*-value
Left femoral fat percentage	−0.45 (−0.55, −0.34)	<0.001
Abdominal fat percentage	−0.43 (−0.58, −0.29)	<0.001

Models adjusted for age at the time of the scan, sex, current smoking status, excessive alcohol use, current glucocorticoid therapy, RA, family history of fracture, BMI and major osteoporotic fractures.

### Sensitivity analysis

When running the same models using complete cases, we observed the same overall pattern: there was no association between RUC and fragility fractures, while rural participants continued to show lower odds of osteoporosis and reduced regional adiposity. The magnitudes of these effects was also similar to those observed in the main analysis. These results are presented in Supplementary Tables S2–S4, available at *Rheumatology Advances in Practice* online.

## Discussion

To our knowledge, this is the first UK-based clinical study to investigate urban/rural differences in BMD, body composition and fragility fracture odds. We found that individuals residing in rural areas had significantly lower odds of being diagnosed with osteoporosis at both the femoral neck and lumbar spine. They also had more favourable body composition profiles, including reduced abdominal and femoral fat percentages. However, these skeletal advantages did not translate into reduced odds of reporting a previous major osteoporotic or hip fracture when compared with urban residents.

Our findings are consistent with some prior studies conducted in high-income countries. The Norwegian Epidemiologic Osteoporosis Studies reported higher distal forearm BMD in rural populations [13] along with the South Dakota study [7], which reported high femoral bone density. However, even among developed nations, differing findings have been observed, with Australian studies highlighting reduced bone density in rural populations [14]. Furthermore, a Swedish study demonstrated a 4-fold higher fracture incidence among urban women ages 60–70 years compared with those in rural areas [15]. Hence it is clear that the urban/rural designation even within developing countries varies significantly, with our research suggesting decreased odds of an osteoporosis diagnosis.

Several environmental and lifestyle-related factors may account for the rural advantage in bone health observed in our study. Air pollution, particularly exposure to fine particulate matter (PM2.5), is higher in urban areas and has been associated with lower BMD and increased osteoporosis risk [16–18]. Research has also suggested that rural residents may have a more active lifestyle that could be advantageous for bone density [19]. Additionally, previous studies have suggested that urbanisation may be linked to lower vitamin D levels, potentially reflecting the higher prevalence of obesity in urban environments, which is itself associated with vitamin D deficiency [20]. Given that our data also showed rural residents have lower body fat, this may support previous research suggesting that these mechanisms may improve bone density in rural populations.

The absence of a significant difference in fracture odds between urban and rural populations in our study may also reflect the structure of the UK healthcare system. Unlike countries such as Australia [14] or the USA [21], where rural residents often face substantial barriers to accessing specialist or hospital care, the UK’s relatively compact geography and centralized National Health Service likely facilitate more equitable access across regions [9]. Although rural areas in the UK do experience health inequalities, including further distances to general practices [22], poorer outcomes for conditions such as cardiovascular disease [23] and challenges in transportation [24], these disparities are generally less pronounced than in countries with more isolated rural populations [25]. This is further supported in that our study had a relatively equal split between urban and rural participants, even though rural participants comprise ≈20% of the population. Hence, proactive screening of the rural population may help explain the lack of an urban/rural difference in fracture odds observed in our cohort. Furthermore, previous research suggests that socio-economic deprivation may play a more important role in determining fracture risk [26, 27] than geographic location alone [9], which further supports our findings.

Our study cohort included a high proportion of rural participants, likely due to the geographic catchment area of our regional DXA scanner in northwest England. This allowed for adequately powered comparisons between urban and rural populations. Given that rural populations are often underrepresented in osteoporosis research, our findings offer a novel insight showing that rural residence in the UK may be associated with higher bone density. However, it is important to acknowledge that these results may not be generalisable to the broader population, as our cohort was drawn from individuals referred for hospital-based assessment. Consequently, it could be the case that the rural population who attend DXA scans are fundamentally different from the general rural population and hence could be a form of selection bias. For example, prior research has suggested that when mobile DXA scanning was used to selectively screen remote populations the FRAX score increased from 3% to 5.7% for hip fractures [28]. This may suggest that the rural population could potentially be less likely to attend scans and thus why no differences in fracture odds were seen in our study. Hence, to determine the true relationship between rural living and bone health, further research using a more representative, longitudinal, population-based sample is necessary to confirm or disprove our findings. Furthermore, despite better bone density but no difference in fragility fracture odds, mediation analysis is needed to understand the relationship between bone density and fracture risk in rural cohorts in the UK.

### Strengths and limitations

A strength of our study is the large number of patients analysed, including the adjustment of confounders. Additionally, the use of imputation strengthens our findings by helping us account for bias arising from missing data.

Limitations include the use of a referred clinical cohort that does not necessarily generalise to the general population. The use of 2011 census data was appropriate for our study period but may not reflect more recent changes in urbanisation or population movement. Our cohort was also predominantly Caucasian, limiting generalisability to more ethnically diverse populations. Finally, the cross-sectional nature of our analysis prevents any causal inferences from being made.

## Conclusions

In conclusion, rural residents in our UK-based cohort exhibited better BMD and more favourable body composition compared with their urban counterparts, but this did not lead to a significant difference in the odds of patients reporting a major osteoporotic fracture. Our results add to the global literature on urban/rural differences in bone health as well as providing UK-specific results for the first time. Further research using nationally representative, longitudinal cohorts is warranted to confirm our findings.

## Supplementary Material

rkag004_Supplementary_Data

## Data Availability

Data will be provided upon reasonable request to the corresponding author.
