# Supplementary material for: Investigating the association between urban rural classification with fragility fractures and bone density in the UK: evidence from a large observational clinical cohort
Source: Rheumatol Adv Pract. 2026 Jan 8;10(1):rkag004. doi: 10.1093/rap/rkag004 (PMC12867579; doi:10.1093/rap/rkag004)
Supplement: rkag004_Supplementary_Data [file rkag004_supplementary_data.docx]

**Supplementary file**

**Supplementary Table S1:** Baseline demographics compared between patient with missing urban rural status vs non-missing urban rural status. Geographic data refers to urban rural status. Table compares all participants scanned.


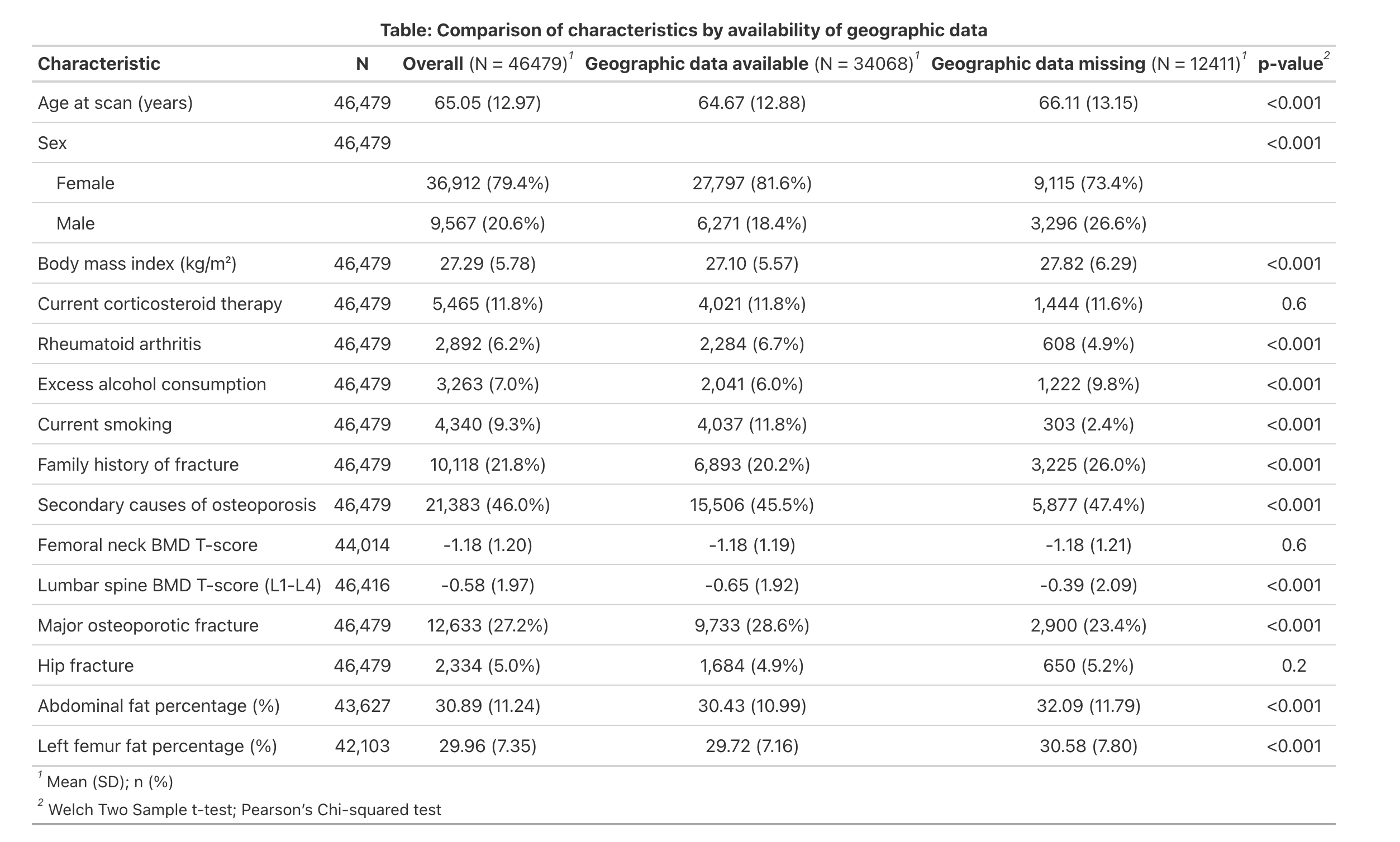


**Complete case analysis results by different outcome:**

*Fragility fracture outcome*

**Supplementary Table S2:** Complete case analysis using logistic regression. Models adjusted for: age at the time of scan, sex, current smoking status, excessive alcohol use, current glucocorticoid therapy, rheumatoid arthritis, family history of fracture, body mass, and femoral neck T-score.

|  | Adjusted urban rural models | p-value |
| --- | --- | --- |
| Major osteoporotic fracture | 0.98 (95% CI 0.93, 1.03) | 0.481 |
| Hip fractures | 0.95 (95% CI 0.84, 1.08) | 0.434 |

*Osteoporosis outcome*

**Supplementary Table S3.** Complete case analysis for osteoporosis models using logistic regression. Models adjusted for: age at the time of scan, sex, current smoking status, excessive alcohol use, current glucocorticoid therapy, rheumatoid arthritis, family history of fracture, BMI and major osteoporotic fracture.

|  | Adjusted urban rural models | p-value |
| --- | --- | --- |
| Femoral neck osteoporosis | 0.81 (95% CI 0.75, 0.87) | <0.001 |
| Lumbar spine osteoporosis | 0.83 (95% CI 0.78, 0.88) | <0.001 |

*Body composition outcome*

**Supplementary Table S4.** Complete case analysis for body composition models using linear regression. Models adjusted for: age at the time of scan, sex, current smoking status, excessive alcohol use, current glucocorticoid therapy, rheumatoid arthritis, family history of fracture, BMI and major osteoporotic

|  | Adjusted urban rural models | p-value |
| --- | --- | --- |
| Femoral Fat percentage | -0.66 (95% CI -0.77, -0.55) | <0.001 |
| Abdominal fat percentage | -0.58 (95% CI -0.76, -0.41) | <0.001 |
